# The Influences of Health Insurance and Access to Information on Prostate Cancer Screening among Men in Dominican Republic

**DOI:** 10.1155/2016/7284303

**Published:** 2016-03-10

**Authors:** Joseph Kangmennaang, Isaac Luginaah

**Affiliations:** ^1^Department of Geography and Environment, University of Waterloo, 200 University Avenue West, Waterloo, ON, Canada N2L 3G1; ^2^Department of Geography, Western University, 1151 Richmond Street, London, ON, Canada N6A 5C2

## Abstract

*Objectives.* Although research demonstrates the public health burden of prostate cancer among men in the Caribbean, relatively little is known about the factors that underlie the low levels of testing for the disease among this population.* Study Design.* A cross-sectional study of prostate cancer testing behaviours among men aged 40–60 years in Dominican Republic using the Demographic and Health Survey (2013).* Methods.* We use hierarchical binary logit regression models and average treatment effects combined with propensity score matching to explore the determinants of prostate screening as well as the average effect of health insurance coverage on screening. The use of hierarchical binary logit regression enabled us to control for the effect of unobserved heterogeneity at the cluster level that may affect prostate cancer testing behaviours.* Results.* Screening varied significantly with health insurance coverage, knowledge of cholesterol level, education, and wealth. Insured men were more likely to test for prostate cancer (OR = 1.65, *p* = 0.01) compared to the uninsured.* Conclusions.* The expansion and restructuring of Dominican Republic universal health insurance scheme to ensure equity in access may improve health access that would potentially impact positively on prostate cancer screening among men.

## 1. Introduction

Dominican Republic (DR) is suffering from the double burden of disease as infectious diseases remain whilst noncommunicable diseases have witnessed an increased share of the burden of diseases [1–3]. For instance, mortality due to noncommunicable causes already accounts for a considerably higher proportion of deaths than communicable causes, rising to 73% of diagnosed deaths in 2005 [1, 4]. Of these noncommunicable diseases, prostate cancer is the leading cause of cancer related deaths in the DR and the Caribbean at large [5, 6]. Globally, an estimated 1.1 million cases of prostate cancer were diagnosed in 2012 and it accounts for 15% of all cancers in men, with an age standardized incidence rates of 79.8 per 100,000 in the Caribbean [5, 7]. It is also the second most common cause of cancer and the sixth leading cause of cancer death among men worldwide and the burden is expected to grow to 1.7 million new cases and 499,000 deaths by 2030 mainly due to the growth and aging of the global population [8, 9]. The highest estimated mortality rates tend to be witnessed mostly in the low- to medium-resource regions of South America, the Caribbean, and Sub-Saharan Africa [8, 9]. An important factor in these global disparities in incidence rates and trends is due to the lack of diagnostic facilities and prostate-specific antigen (PSA) testing in most low-resource settings [10].

Even though prostate cancer is the major cancer in men of African and Caribbean descent throughout the world, incidence and mortality rates are often underestimated [11] largely because testing is believed to be uncommon [3, 12, 13]. The high cost of materials, inefficient health care delivery systems, and lack of skill workers may also undermine testing. For instance, Simard and Jemal report that a substantial proportion of cancer related infections in Low and Middle Income Countries (LMIC) are preventable through the appropriate public health intervention [12]. Also, increasing burdens of noncommunicable diseases (NCDs) have been observed among people with lower socioeconomic status among the poor and less-educated [14–17]. Despite the increasing burden of prostate cancer and the availability of low cost services such as the use of human papillomavirus (HPV) and PSA [12] screening tools, testing for the purpose of early detection of cases remains low in most poor resource settings. Prostate cancer screening could reduce significantly the rate of death [18], risks of metastasis, and tumor progression if cases are detected early [19]. Early testing has been acknowledged as contributing to the steady trends in prostate cancer rates observed in countries with higher rates of PSA testing including the United States and Canada [20–22]. However, testing for prostate cancer in the Caribbean and other poor resource settings remains low [23]. Compounding the lower testing levels are barriers related to access to cancer related health care, including diagnosis and treatment, and the poor health infrastructure in most of these poor resource settings [6, 24].

In the DR, prostate cancer accounts for 43% of all cancer cases and 37% of all cancer mortality among adult men and has a 5-year prevalence rate of 55.1 per 100,000 [5]. Despite increasing prevalence, prostate cancer continues to receive low public health priority in Dominic Republic (DR) and the Caribbean at large [3]. Current evidence indicates that the factors that contribute to the low levels and disparities in screening largely stem from differences in health care access [14–16, 25–27], access to knowledge and information, and the lack of early detection services [6, 23]. Furthermore, inadequate public health infrastructure and other competing health problems such as HIV/AIDS seem to account for the rather low policy attention presently given to prostate cancer [23, 28]. In the United States and Canada, health insurance coverage has also been shown to be associated with early detection of cancer cases [20, 24, 29] and may reflect the higher risks of late-stage diagnosis of prostate cancer among uninsured individuals. This alludes to the vital role of health insurance coverage as a major predictor of cancer screening given its enabling effects on health care access and financing. However, in the DR, despite the introduction of a national health insurance in 2008, only 54% of the population are covered under health insurance (29% within the contributory plan, 25% within a subsidized plan) with the rest paying out of pocket for health services [2].

Even though the risk factors for prostate cancer among men are still contested, older age, black race/ethnicity, and heredity have been associated with higher risk of developing the disease [8, 30]. The few studies from the Caribbean region suggest that there is a general lack of public awareness about prostate cancer and associated risk factors rooted in the failure of the health care system to promote testing for the disease [23]. This study examines the influence of health insurance coverage, access to knowledge, and information on men decision to screen for prostate cancer in the DR. The findings we hope will be of significant policy directions for the Caribbean in general and for the DR government in particular as it strives to improve screening for prostate cancer to reduce the associated burden.

## 2. Methods

### 2.1. Data and Sample

#### 2.1.1. Data

This study used the 2013 Dominican Republic Demographic and Health Survey (DRDHS, 2013). The Demographic and Health Survey is a nationally representative dataset collected jointly by the National Statistical Bureau and Ministry of Health of Dominican Republic and MEASURE DHS program in Calverton, Maryland, USA. The DRDHS is collected periodically to provide data on national demographics and health indicators to policy makers, planners, and researchers and has recently introduced a set of indicators on prostate cancer screening. The current study focuses on 3,272 men aged between 40 and 60 years. Ethics was granted by the host country, Macro (a US-based organization that collaborates and provides technical assistance to DHS) and other implementing partners.

#### 2.1.2. Measures

**Figure 1:**
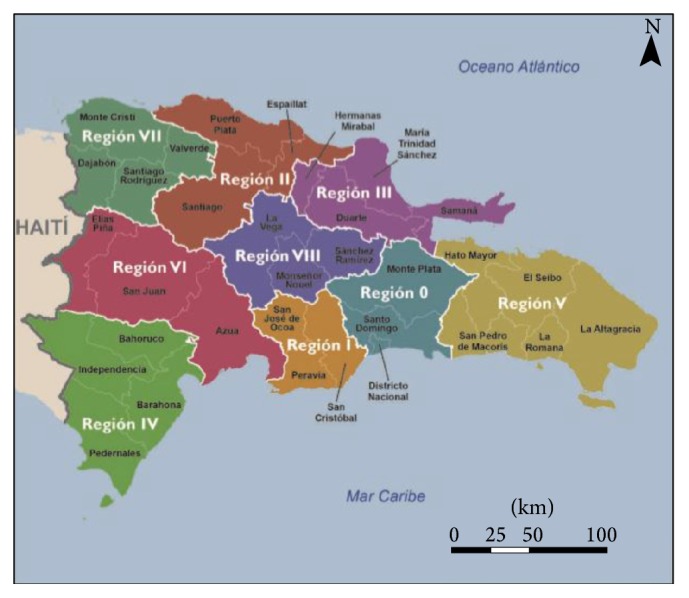
Regional map of Dominican Republic, adopted from the DRDHS, 2013.

The dependent variable of this study, prostate cancer screening, was constructed from the question, “Have you ever done a prostate exam?” Respondents indicated whether they have done the exam or not. Thus, the variable is dichotomously coded “0” for men who haven't undergone the procedure and “1” for men that have been examined. Our focal independent variable, health insurance coverage, was constructed from the question, “Are you covered by health insurance” coded (0 = not covered; 1 = covered). To assess the effect of risk factors, the study included a variable on whether respondents have been advised by a doctor about their cholesterol level (0 = no; 1 = yes) and also included a variable on smoking (0 = no; 1 = yes) given that prior literature have indicated these as potential risk factors for developing prostate cancer [30]. To capture the effect of health literacy, the study also included a variable on education (0 = no education; 1 = primary; 2 = secondary; and 3 = higher) and whether households have received information on prostate cancer prevention in the last 12 months (0 = no; 1 = yes). The role of health literacy was further examined by a variable tapping into exposure to media in which men were asked as to whether they listen to radio (0 = not at all; 1 = often; and 2 = very often), watch television (0 = not at all; 1 = often; and 2 = very often), and read newspapers (0 = not at all; 1 = often; and 2 = very often). The analysis also examined the mediating effect of socioeconomic variables using wealth status, and a composite index based on the household's ownership of a number of consumer items, assets, and the characteristics of the dwelling was constructed using principal component analysis. Principal component analysis (PCA), a technique for extracting from a set of variables an orthogonal linear combination of the variables that capture the common information most successfully, was used to construct an overall index of household wealth [31, 32]. Each asset was normalized by its mean and standard deviation and dummied into quintiles (0 = poorest; 1 = poorer; 2 = middle; 3 = richer; and 4 = richest). Demographic variables included in the analysis are age of respondents in 5-year categories (0 = 40–44; 1 = 45–49; 2 = 50–54; 3 = 55–60), marital status (0 = never married; 1 = married; 2 = separated), place of residence (0 = urban; 1 = rural), and the religious denomination of respondents (0 = Catholics; 1 = other; 2 = no religion). The DRDHS used the regional classification of the Ministry of Health (MOH) to characterise the geographic region of residence (0 = 0; 1 = i; 2 = ii; 3 = iii; 4 = iv; 5 = v; 6 = vi; 7 = vii; 8 = viii). Kindly see Figure 1 for what constitute each region. The variables were grouped under three main categories in our models: (1) health insurance and risk variables; (2) knowledge and information variables; and (3) demographic and socioeconomic factors.

### 2.2. Analytical Strategy

We used binary logistic regression to analyze our dependent variable given its binary nature. Logit models are built under the assumption of independence of respondents, but the DRDHS has a hierarchical structure with respondents nested within survey clusters which could possibly bias our standard errors. To control for biases in parameter estimates and to evaluate the impact of cluster variables on testing for prostate cancer, a random effects regression analysis was employed using GLLAMM in Stata (also see [33–35]). Sample characteristics of our dependent and some selected variables are depicted in Table 1.  Table 2 presents our bivariate associations between the predictor variables and prostate cancer screening. This is followed by three multivariate hierarchical regressions models. We further conducted average treatment effects of health insurance coverage on prostate cancer testing using different propensity score matching techniques. In our study, the insured were men with health insurance whilst the counterfactual group were those without insurance coverage. Under ideal conditions, the effective strategy would be to obtain the average effect of insurance coverage on prostate cancer screening, also known as the average treatment effect (ATE). ATE can be expressed as

(1) $$\begin{array}{c} ATE=E\left(\;NY_{i}\;\right)=E\left(\;Y_{i,1}-Y_{i,0}\;\right), \end{array}$$

where NY_*i*_ refers to the effect of insurance coverage on screening, *E* is expected value, and *Y*_*i*,1_ is the value for the insured whilst *Y*_*i*,0_ is the value for the uninsured group. At least one of the outcomes (either insured or not) is observed whilst the other is not observed for each individual; thus every man is either insured or not. We therefore adopted augmented inverse probability weighting, nearest neighbor matching, and kernel-based propensity score matching to create the unobserved component (counterfactual group) [36–40]. Becker and Ichino provides detailed description of these matching methods [41]. The* teffects* command available in STATA13 was used to build the average treatment models.

## 3. Results

Notably, 31% of men reported ever being examined for prostate cancer whilst about 62% of them reported being covered under health insurance. Fifty-nine percent of households indicated receiving information on prostate cancer prevention from a health worker in the last 12 months. The majority of men had only primary education, were currently married, were residing in urban areas, are Catholics, and had access to both print and electronic media. Also, about 27% of men were in the poorer wealth category.

### 3.1. Bivariate Results

Results from the bivariate logistic models are reported in Table 2. Men covered by health insurance (OR = 2.12, *p* = 0.01) were more likely to be examined for prostate cancer. Men in households that reported receiving information on prostate cancer prevention (OR = 1.38, *p* = 0.01) were more likely to test for prostate cancer than men in households that did not receive information on prostate cancer prevention. Compared to men who did not know their cholesterol levels, those who were advised by a doctor about their cholesterol level (OR = 4.20, *p* = 0.01) were more likely to report testing for prostate cancer. Also, compared to men with no education, those with primary (OR = 2.02, *p* = 0.01), secondary (OR = 2.79, *p* = 0.01), and tertiary (OR = 7.27, *p* = 0.01) education were more likely to report testing for prostate cancer. Men with access to information through the media, listening to radio, watching television, and reading newspapers were more likely to report testing for the disease. Among the sociocultural factors, compared to urban men, men dwelling in rural areas were less likely (OR = 0.76, *p* = 0.01) to test for prostate cancer while residents in region IV (OR = 0.64, *p* = 0.01) were also less likely to test for prostate cancer compared to region I. Increasing levels of wealth were associated with men testing for prostate cancer; poor (OR = 1.39, *p* = 0.01), middle (OR = 2.19, *p* = 0.01), richer (OR = 3.49, *p* = 0.01), and richest wealth quintiles (OR = 6.41, *p* = 0.01) were more likely to test for prostate cancer compared to the poorer category. These bivariate analyses were useful in revealing which variables to include in our multivariate analysis.

### 3.2. Multivariate Analysis

The multivariate models are shown in Table 3. In the first model we estimated the effects of health insurance coverage and risk factors on testing behaviours, the second model controls for the effect of knowledge and information on testing, and the third model controls for demographic and socioeconomic variables. The final model estimated interaction effects between insurance coverage, wealth, and place of residence.

In model 1, compared to uninsured men, those under insurance coverage (OR = 1.91, *p* = 0.01) were more likely to be examined for prostate cancer. Also, men who were told of their cholesterol level by a doctor (OR = 3.85, *p* = 0.01) were more likely to report testing; however men who reported smoking cigarettes (OR = 0.49, *p* = 0.01) were less likely to be screened for prostate cancer. In model 2, we found that households that received information on prostate cancer prevention (OR = 1.22, *p* = 0.05) and had access to information through reading newspapers very often (OR = 1.37, *p* = 0.01) were associated with higher likelihood of being examined for prostate cancer compared to those with no information on prostate cancer. Similarly, men with primary (OR = 1.48, *p* = 0.01), secondary (OR = 1.75, *p* = 0.01), and tertiary (OR = 3.68, *p* = 0.01) education were more likely to report testing compared to men with no education. These results suggest that increased exposure to health information is an important determinant of men decision to seek prostate cancer examination. Furthermore, even after controlling for knowledge and information variables, the association between testing and health insurance coverage remained robust.

Turning to demographic and socioeconomic variables, we found that age of respondent, marital status, region of residence, and wealth were significant predictors of screening for prostate cancer. Compared to never married men, married (OR = 3.10, *p* = 0.01) and separated (OR = 2.37, *p* = 0.01) men were more likely to test for prostate cancer. Residents of region I (OR = 1.47, *p* = 0.05), region II (OR = 1.84, *p* = 0.01), region III (OR = 1.71, *p* = 0.01), region VI (OR = 1.60, *p* = 0.05), and region VII (OR = 1.87, *p* = 0.01) were all more likely to test compared to residents of region O. In contrast to the poorest category, men in the middle (OR = 1.56, *p* = 0.01), richer (OR = 2.35, *p* = 0.01), and richest (OR = 3.23, *p* = 0.01) categories were all more likely to test for prostate cancer. In model IV, we examined whether insurance coverage interacting with higher levels of wealth and place of residence are associated with prostate cancer testing. The results show that men who are uninsured and of middle wealth (OR = 1.73, *p* = 0.01), uninsured and rich (OR = 2.48, *p* = 0.01), uninsured and richest (OR = 3.46, *p* = 0.01), insured and middle (OR = 1.47, *p* = 0.01), insured and rich (OR = 2.27, *p* = 0.01), and insured and richest (OR = 3.04, *p* = 0.01) were more likely to test for prostate cancer compared to uninsured and poorer men. No statistically significant cross-level interactions effects were found between place of residence and insurance coverage on prostate cancer screening.

The association whereby insured persons were more likely to screen for prostate cancer could be due to positive selection bias (see [38, 40, 42]), implying that men who are more educated, are wealthy, live in urban centres, and are unhealthy might be more likely to enrol in health insurance which might impose biases in our analysis. In order to correct these problems, we derived the average treatment effects, employing propensity score matching techniques including kernel-based propensity score matching, nearest neighbor, and augmented inverse probability weighting to control for possible selection biases [37, 39, 40]. Propensity score matching enabled us to construct a statistical comparison group by matching every individual covered by health insurance with an observation with similar characteristics from the group of individuals without insurance coverage. The average treatment effects of insurance coverage on prostate cancer screening from the various matching techniques are as follows: augmented inverse probability weighting (*β* = 0.083, *p* = 0.01), kernel-based propensity scores (*β* = 0.097, *p* = 0.01), and nearest neighbor matching (*β* = 0.079, *p* = 0.01) depict positive effects of insurance coverage on prostate cancer screening even after accounting for other covariates (see Tables 4, 5, and 6). The results imply that being insured increases prostate cancer screening by 0.083, 0.097, and 0.079 index points under augmented inverse probability weighting, kernel, or nearest neighbor matching, respectively.

## 4. Discussion

We examined the factors associated with prostate cancer screening among men in Dominican Republic. Our study reveals a robust relationship between health insurance coverage and screening for prostate cancer and posits the significant role of health care access on men attitude to screen for prostate cancer in this context. Prior studies have reported wide disparities in health care utilization in the DR, entrenched in health care access and income inequalities, including in the area of cancer testing [6, 23, 28]. These studies have shown that over 56% of Dominicans rely on out-of-pocket payment and poor-quality health services provided by an already overburdened and underfinanced public health system. Only a minority of Dominicans enjoy quality services from private facilities that primarily serve high income groups, leaving the majority of health seekers to rely on public hospitals. Even among those covered under health insurance, the plan only provides limited coverage for cancer diagnostics and treatment [28]. Universal health insurance coverage with a pro-poor focus may help reduce out-of-pocket health expenses [43–46], reduce inequalities in access, increase the frequency and quality of men interacting with health workers, and also encourage men to undertake preventive services, including screening for prostate cancer and others at risk factors such as high cholesterol levels. However, it is important to acknowledge that, in its current formulation, insurance coverage would not lead to equitable access to prostate cancer screening as it favours only salaried workers and the rich. A further interaction effect analysis (wealth levels and insurance coverage) revealed that insured poor and uninsured poor were not statistically different from uninsured poorer group and were all less likely to report testing. Policy attention should be given to this group (poorer and poor men) as the literature suggests they are most vulnerable to noncommunicable diseases [15, 16]. There is the need to ensure synergies between the two separate schemes, the contributory and subsidized schemes to guarantee that both scheme beneficiaries have equitable access to quality health care.

Similarly, as expected, our results also revealed that household wealth played significant role in men's decision to seek prostate cancer screening. Compared to men from poorer households, those from middle, richer, and richest households were more likely to screen for prostate cancer. The richer and richest categories were more likely to report testing for the disease, reemphasising the notion that it is mostly those who have the financial means to obtain the contributory insurance that overcome barriers to accessing health care. As the interaction effects reveal, it is mostly the rich and richer men that are likely to seek screening and this again reemphasizes the argument that it is mostly the rich men who have access to insurance coverage. The relatively low likelihood of testing among the poor highlights the issue of socioeconomic inequalities to cancer screening. Since testing is a gateway to treatment, the findings of this study also suggest potential socioeconomic disparities in morbidity and mortality from cancer in Dominican Republic as reported in other developing countries [15, 16, 25].

The positive relationship between knowledge and information access and screening for prostate cancer is worth noting and consistent with studies that have found that health seekers who have regular contact with health professionals tend to be better informed about health issues and are more likely to receive advice [47, 48]. The significant relationship between men who were told of their cholesterol level and those who receive information on preventive measures and screening for prostate cancer reflects more affirmative attitudes toward learning about prostate cancer and engaging in healthy behaviours that would lead to early detection and prevention of the disease [49]. A possible explanation for this finding is that insured men may be more likely to interact with health professionals as they face less financial barriers in accessing health care and hence may be more likely to know their cholesterol level and adopt positive health behaviours. However even after accounting for interaction effects of health insurance with other factors (see Table 3), the association between knowledge of cholesterol level and prostate cancer testing remained significant implying that knowledge of one's cholesterol level may have an independent effect on prostate cancer screening. Our results also show that men with higher levels of education or exposure to health information through newspapers were more likely to screen for prostate cancer. This is largely consistent with others that hold the view that information which may include specific facts about a disease is vital to a person's decision about health-related action [49]. Similarly, increasing categories of age were associated with higher likelihood of testing. This also reflects positive attitudes as aging has been indicated as one of the potential risk factors for developing prostate cancer. These findings draw attention to the need for increased emphasis on awareness promotion in order to equip the public about specific facts and risk factors of prostate cancer and to encourage men to undertake screening.

Economically, Dominican Republic is a middle income country and the largest economy in Central America and the Caribbean. However, there exists marked inequality in income distribution which may further compound the question of equitable access to health services especially in areas such as region O (National District) [3, 28, 50]. This likely explains why men from this region were uniquely less likely to screen for prostate cancer. Furthermore, even though prostate cancer screening is generally low in Dominican Republic, the findings of this study suggest the existence of wide geographical variations in terms of screening.

Even though this study provides valuable insights into the determinants of prostate cancer screening in Dominican Republic, this study is not without some limitations. First, as with all cross-sectional datasets, we were unable to make causal linkages between our explanatory variables and prostate cancer screening. Also, some biases may have been introduced in our data as respondents will most likely provide socially acceptable responses and the DRDHS could not physically validate these responses. For instance, household wealth, which is a composite index of a household ownership of asset and characteristics of household dwelling unit, is particularly prone to response biases as most people may not remember all their assets. Such a measure of wealth does not also take into account the household dynamics such as intrahousehold distribution of resources and the power dynamics that operate at the household level. Despite these challenges, in a context where most people either cannot estimate their annual income or may refuse to disclose such amounts, using an index of ownership of assets and housing characteristics is an innovative way to obtain a measure of socioeconomic status. Even though the use of propensity score matching might help correct the bias, due to problems of simultaneous causality and the cross-sectional nature of our data, we were unable to establish causal links between insurance coverage and screening [51, 52]. However establishing causality is not the main intention of this paper as we were mainly interested in understanding the impact of health insurance on prostate cancer screening. Hence, in the absence of ideal randomized trials, propensity score matching methods offer an opportunity to use observational data to established treatment effects. Nonetheless, the findings are important to scale up prostate cancer screening in Dominican Republic as well as in other Caribbean countries that are at a greater risk of prostate cancer.

## 5. Conclusion

In conclusion, this paper has examined factors associated with screening for prostate cancer among men in Dominican Republic with particular emphasis on the role of health insurance and access to information. The potential role played by health insurance coverage in determining screening as shown by this study signals the need for an equitable universal health insurance scheme that ensures equitable access to improved health services. In addition, the findings of this study suggest the need to reinforce policies and programs that promote access to information and knowledge about risk factors and encourage men to frequently undertake preventive services such as checking their cholesterol levels. Routine screening clinics for underprivileged men and utilizing mobile health care workers to serve poor communities will prove useful to curbing prostate cancer incidence. Extra efforts have to be made to target uneducated men through the use of local dialects in public health care prevention messages with a focus on making known the various risk factors of prostate cancer. The use of community-based health workers may be an option to promote information that will increase the uptake of prostate cancer screening with such information focusing on the benefits of early detection. However, our findings suggest that such initiatives may have the greatest impact on men especially in region O. Where feasible, cancer control programs should be integrated with already established disease control programs.

## Highlights

- Only 31% of Dominican Republic men reported ever testing for prostate cancer.
- Men covered under health insurance were more likely to report testing for prostate cancer compared to uninsured men.
- Men that were informed by a doctor about their cholesterol level and had primary education or above were more likely to report screening than their counterparts that were not aware of their risk levels and had no education. We advocate that men should be encouraged to adopt positive attitudes such as routine medical check-up.
- We advocate for the adoption of a universal health insurance scheme that ensures equity in access to health care, incorporate lessons and experiences of similar schemes in other countries, and extend insurance coverage for informed prostate cancer diagnostics and treatment.

## Figures and Tables

**Table 1:** Descriptive statistics.

Variables	Frequency (%)
Prostate cancer testing	
No	69.35
Yes	30.65
Health insurance	
No	38.33
Yes	61.67
Being told by a doctor that your cholesterol level is high	
No	41.01
Yes	58.98
Received information on prostate cancer prevention	
No	40.98
Yes	59.02
Education	
None	8.59
Primary	57.33
Secondary	22.71
Tertiary and higher	11.37
Listen to radio	
None	6.69
Often	15.83
Very often	77.48
Watch television	
None	5.26
Often	12.35
Very often	82.40
Read newspapers	
None	39.45
Often	19.82
Very often	40.73
Age of respondent (mean)	49
Marital status	
Never married	5.81
Married	71.49
Separated	22.71
Religion	
Catholic	57.61
Other Christians	15.50
No religion	26.88
Region of residence	
0	12.63
I	10.67
II	12.16
III	11.34
IV	8.99
V	10.67
VI	10.88
VII	10.82
VIII	11.86
Place of residence	
urban	66.29
Rural	33.71
Wealth	
Poorer	27.32
Poor	19.71
Middle	19.53
Richer	17.57
Richest	15.86
Observation	**3,272**

**Table 2:** Bivariate associations between independent variables and prostate cancer screening.

Prostate cancer testing	OR (95% confidence interval)
Health insurance (ref: no)	
Yes	2.12 (1.784–2.512)^*∗∗∗*^
Cholesterol knowledge (ref: no)	1.00
Yes	4.20 (3.293–5.359)^*∗∗∗*^
Smokes cigarettes (ref: no)	1.00
Yes	0.45 (0.354–0.580)^*∗∗∗*^
Received information on prostate cancer prevention (ref: no)	1.00
Yes	1.38 (1.175–1.623)^*∗∗∗*^
Education (ref: none)	1.00
Primary	2.02 (1.427–2.863)^*∗∗∗*^
Secondary	2.79 (1.923–4.016)^*∗∗∗*^
Tertiary and higher	7.27 (4.774–10.536)^*∗∗∗*^
Listen to radio (ref: none)	1.00
Often	1.27 (0.864–1.87)
Very often	1.56 (1.106–2.187)^*∗∗∗*^
Watch television (ref: none)	1.00
Often	1.48 (0.937–2.367)
Very often	1.97 (1.295–2.941)^*∗∗∗*^
Age of respondent (ref: 40–44)	1.00
45–49	1.88 (1.497–2.364)^*∗∗∗*^
50–54	2.73 (2.159–3.455)^*∗∗∗*^
55–60	4.32 (3.403–5.508)^*∗∗∗*^
Marital status (ref: never married)	1.00
Married	4.89 (3.011–7.956)^*∗∗∗*^
Separated	2.71 (1.626–4.499)^*∗∗∗*^
Religion (ref: Catholic)	1.00
Other Christians	0.94 (0.752–1.176)
No religion	0.69 (0.572–0.838)^*∗∗∗*^
Region of residence (ref: 0)	1.00
I	1.07 (0.742–1.535)
II	1.29 (0.908–1.821)
III	1.28 (0.904–1.838)
IV	0.64 (0.429–0.957)^*∗∗*^
V	0.87 (0.606–1.262)
VI	0.85 (0.588–1.231)
VII	1.18 (0.825–1.694)
VIII	0.95 (0.667–1.367)
Place of residence (ref: urban)	1.00
Rural	0.76 (0.625–0.923)^*∗∗∗*^
Wealth (ref: poorer)	1.00
Poor	1.39 (1.069–1.808)^*∗∗∗*^
Middle	2.19 (1.700–2.817)^*∗∗∗*^
Richer	3.49 (2.708–4.498)^*∗∗∗*^
Richest	6.41 (4.918–8.346)^*∗∗∗*^

Standard errors in parentheses ^*∗∗∗*^ *p* = 0.01, ^*∗∗*^ *p* = 0.05.

**Table 3:** Determinants of testing for prostate cancer using hierarchical logistic regression.

Variable	Model (1)	Model (2)	Model (3)	Model (4)
	OR (SE)	OR (SE)	OR (SE)	OR (SE)
Health insurance (ref: no)	1.00	1.00	1.00	1.00
Yes	1.91 (0.171)^*∗∗∗*^	1.73 (0.156)^*∗∗∗*^	1.64 (0.157)^*∗∗∗*^	1.81 (0.417)^*∗∗*^
Risk factors				
Cholesterol level (ref: no)	1.00	1.00	1.00	1.00
Yes	3.85 (0.489)^*∗∗∗*^	3.37 (0.433)^*∗∗∗*^	3.02 (0.412)^*∗∗∗*^	2.90 (0.384)^*∗∗∗*^
Do you smoke (ref: no)	1.00	1.00	1.00	1.00
Yes	0.49 (0.064)^*∗∗∗*^	0.54 (0.071)^*∗∗∗*^	0.52 (0.072)^*∗∗∗*^	0.52 (0.071)^*∗∗∗*^
Access to information				
Received information on cancer prevention (ref: no)		1.00	1.00	1.00
Yes		1.22 (0.106)^*∗∗*^	1.05 (0.097)	1.05 (0.094)
Education (ref: none)		1.00	1.00	1.00
Primary		1.48 (0.282)^*∗∗*^	1.67 (0.336)^*∗∗*^	1.67 (0.330)^*∗∗∗*^
Secondary		1.75 (0.364)^*∗∗∗*^	1.88 (0.418)^*∗∗∗*^	1.87 (0.411)^*∗∗∗*^
Tertiary and higher		3.68 (0.832)^*∗∗∗*^	3.12 (0.764)^*∗∗∗*^	3.02 (0.727)^*∗∗∗*^
Listen to radio (ref: none)		1.00	1.00	1.00
Often		1.07 (0.234)	1.11 (0.256)	1.13 (0.256)
Very often		1.05 (0.197)	1.02 (0.203)	1.02 (0.199)
Watch television (ref: none)		1.00	1.00	1.00
Often		1.14 (0.296)	1.11 (0.302)	1.088 (0.287)
Very often		1.21 (0.275)	1.12 (0.267)	1.113 (0.257)
Read newspapers (ref: none)		1.00	1.00	1.00
Often		1.19 (0.145)	1.17 (0.149)	1.17 (0.148)
Very often		1.37 (0.147)^*∗∗∗*^	1.39 (0.162)^*∗∗∗*^	1.38 (0.158)^*∗∗∗*^
Demographic factors				
Age categories (40–44)			1.00	1.00
45–49			1.91 (0.238)^*∗∗∗*^	1.92 (0.233)^*∗∗∗*^
50–54			3.27 (0.425)^*∗∗∗*^	3.26 (0.412)^*∗∗∗*^
55 and over			5.49 (0.750)^*∗∗∗*^	5.43 (0.719)^*∗∗∗*^
Marital status (ref: never married)			1.00	1.00
Married			3.08 (0.804)^*∗∗∗*^	3.01 (0.772)^*∗∗∗*^
Separated			2.36 (0.644)^*∗∗∗*^	2.32 (0.625)^*∗∗∗*^
Religion (ref: Catholic)			1.00	1.00
Other Christians			1.06 (0.135)	1.06 (0.131)
No religion			0.87 (0.095)	0.87 (0.093)
Place of residence (ref: urban)			1.00	1.00
Rural			1.13 (0.123)	Na
Region of residence (ref: 0)				
I			1.46 (0.284)	1.46 (0.265)^*∗∗*^
II			1.83 (0.345)^*∗∗∗*^	1.80 (0.317)^*∗∗∗*^
III			1.68 (0.327)^*∗∗∗*^	1.68 (0.305)^*∗∗∗*^
IV			1.07 (0.232)	1.09 (0.223)
V			1.36 (0.267)	1.36 (0.249)
VI			1.59 (0.318)^*∗∗*^	1.58 (0.294)^*∗∗*^
VII			1.85 (0.360)^*∗∗∗*^	1.84 (0.335)^*∗∗∗*^
VIII			1.32 (0.256)	1.33 (0.241)
Socioeconomic factors				
Wealth (ref: poorest)			1.00	
Poorer			1.12 (0.164)	n/a
Middle			1.55 (0.227)^*∗∗∗*^	n/a
Richer			2.34 (0.356)^*∗∗∗*^	n/a
Richest			3.20 (0.544)^*∗∗∗*^	n/a
Interaction effects				
Insurance coverage and wealth (ref: uninsured and poorest)				1.00
Uninsured and poorer				1.329 (0.322)
Uninsured and middle				1.73 (0.412)^*∗∗*^
Uninsured and richer				2.48 (0.593)^*∗∗∗*^
Uninsured and richest				3.46 (0.907)^*∗∗∗*^
Insured and poorer				1.04 (0.181)
Insured and middle				1.47 (0.255)^*∗∗*^
Insured and richer				2.27 (0.406)^*∗∗∗*^
Insured and richest				3.04 (0.591)^*∗∗∗*^
Uninsured and urban				1.00
Uninsured and rural				1.13 (0.189)
Insured and rural				1.158 (0.143)
Random effects	1.65 (0.126)^*∗∗∗*^	1.50 (0.128)^*∗∗∗*^	1.38 (0.150)^*∗∗∗*^	1.39 (0.147)^*∗∗∗*^
Constant	0.26 (0.021)^*∗∗∗*^	0.09 (0.029)^*∗∗∗*^	0.01 (0.003)^*∗∗∗*^	0.01 (0.003)^*∗∗∗*^
Observations	**3,272**	**3,272**	**3,272**	**3,272**

Standard errors in parentheses ^*∗∗∗*^ *p* = 0.01, ^*∗∗*^ *p* = 0.05.

OR refers to odds ratios and SE refers to standard errors.

Variables included in these models: model 1 = *health insurance and risk factors* (health insurance, told of cholesterol level, and ever smoke cigarettes), model 2 = model 1 + access to information (received information on cancer prevention, education, listen to radio, watch television, and literacy), model 3 = model 2 + demographic factors (age category, marital status, religion, region and place of residence, and wealth), model 4 = model 3 + interaction effects.

**Table 4:** Average effects of insurance coverage using augmented inverse probability weighting.

Potential outcome means	Coefficient (SE)
Insurance coverage	
Uninsured	0.254 (0.012)^*∗∗∗*^
Insured	0.337 (0.0102)^*∗∗∗*^
Average treatment effects	0.083 (0.0115)^*∗∗∗*^
Outcome model parameter estimates (insured men)	
Received information on prostate cancer prevention	−0.288 (0.047)^*∗∗∗*^
Education	0.072 (0.017)^*∗∗∗*^
Age	0.083 (0.010)^*∗∗∗*^
Marital status	0.001 (0.017)
Region of residence	0.025 (0.023)
Place of residence	0.004 (0.004)
Wealth	0.061 (0.009)^*∗∗∗*^
Contact	−0.683 (0.089)^*∗∗∗*^
Outcome model parameter estimates (uninsured men)	
Received information on prostate cancer prevention	0.025 (0.021)
Education	0.068 (0.014)^*∗∗∗*^
Age	0.106 (0.009)^*∗∗∗*^
Marital status	0.002 (0.020)
Region of residence	0.044 (0.022)^*∗∗*^
Place of residence	0.003 (0.004)
Wealth	0.082 (0.008)^*∗∗∗*^
Contact	−0.838 (0.081)^*∗∗∗*^
Parameter estimates for treatment model	
Told of cholesterol level	0.665 (0.137)^*∗∗∗*^
Ever smoke cigarettes	−0.307 (0.100)^*∗∗∗*^
Received information on prostate cancer prevention	0.091 (0.076)
Education	0.168 (0.056)^*∗∗∗*^
Listen to radio	0.050 (0.067)
Watch television	−0.032 (0.076)
Read newspapers	0.117 (0.047)^*∗∗∗*^
Age category	0.091 (0.034)^*∗∗∗*^
Marital status	−0.228 (0.076)^*∗∗∗*^
Religion	−0.011 (0.043)
Region of residence	−0.138 (0.081)^*∗*^
Place of residence	0.022 (0.014)
Wealth	0.051 (0.032)
Constant	−0.387 (0.352)^*∗∗∗*^

^*∗∗∗*^ *p* = 0.01, ^*∗∗*^ *p* = 0.05, and ^*∗*^ *p* = 0.10.

**Table 5:** Average effects of insurance coverage on prostate cancer testing using kernel-based propensity score matching.

Prostate cancer testing	Coefficient	Standard errors	Confidence interval
Insurance coverage (insured versus uninsured)	0.097^*∗∗∗*^	0.0175	(0.062505–0.1309875)

^*∗∗∗*^ *p* = 0.01.

**Table 6:** Average effects of insurance coverage on prostate cancer testing using nearest neighbor propensity score matching.

Prostate cancer testing	Coefficient	Standard errors	Confidence interval
Insurance coverage (insured versus uninsured)	0.079^*∗∗∗*^	0.0179	(0.0438178–0.1143218)

Standard errors in parentheses ^*∗∗∗*^ *p* = 0.01.

Variables included in these models: at risk factors (told of cholesterol level, ever smoke cigarettes), access to information (received information on cancer prevention, education, listen to radio, watch television, and literacy), demographic (age category, marital status, religion, region and place of residence, and wealth).
